# CD147 Expression Is Associated with Tumor Proliferation in Bladder Cancer via GSDMD

**DOI:** 10.1155/2020/7638975

**Published:** 2020-02-20

**Authors:** Junming Peng, Hongtao Jiang, Jinan Guo, Jiansheng Huang, Qian Yuan, Jing Xie, Kefeng Xiao

**Affiliations:** Department of Urology, The First Affiliated Hospital of Southern University of Science and Technology/Shenzhen People's Hospital, Shenzhen, Guangdong 518000, China

## Abstract

**Purpose:**

CD147, also known as BSG, is a type I transmembrane glycoprotein that belonged to immunoglobulin superfamily. Mature CD147 is an N-linked glycosylated protein and exists on the transmembrane and as soluble forms in tumors. However, the function of CD147 in cell proliferation of bladder cancer (BC) remains to be elucidated.

**Methods:**

The study included 159 patients with BC and 68 healthy controls. The expression of CD147 and gasdermin D (GSDMD) was analyzed by immunohistochemistry (IHC). Western blotting was performed to detect the expression of proteins in BC cells. The relationship between CD147 and GSDMD was analyzed by the IHC score.

**Results:**

The expression of CD147 was significantly increased in BC when compared to healthy controls, and the level of CD147 was correlated with tumor proliferation characterized by Ki-67, which is a cell proliferation antigen. In addition, CD147 treatment of BC cells increased the expression of GSDMD, leading to increased Ki-67 expression, while CD147 blockade with peptide in BC significantly reduced GSDMD expression, resulting in reduced cell proliferation. Furthermore, overexpression of GSDMD markedly overcame the inhibitory effect of CD147 peptide on tumor proliferation. BC patients with overexpression of CD147 showed correlation with GSDMD and demonstrated significantly poorer prognosis and overall survival rate.

**Conclusion:**

These findings suggested that high expression of CD147 contributed to tumor proliferation in BC via GSDMD, which might in turn act as an unfavorable prognostic marker.

## 1. Introduction

Bladder cancer (BC) is one of the most common urologic malignancies and is typically classified into two main pathological groups, the nonmuscle invasive bladder cancer (NMIBC) and the muscle invasive bladder cancer (MIBC). The standard treatment for MIBC is neoadjuvant chemotherapy followed by radical cystectomy, but the long-term disease-free survival rate remained low [1, 2]. The time of diagnosis plays a crucial role in achieving a good prognosis. Therefore, determination of new molecular markers that act as potential therapeutic targets and prognostic indicators of BC is vital for conducting a clinically accurate diagnosis and treatment [3, 4].

Emmprin is also known as CD147 or basigin (BSG) and belongs to the superfmaily of human immunoglobulins. Several studies have demonstrated overexpression of CD147 in many malignant tumors, such as breast cancer, lung cancer, and human malignant melanoma, suggesting its role in the promotion of tumorigenesis and metastasis [5]. Reports have showed the involvement of CD147 in cell glycolytic metabolic pathways that enable cancer cells to divide and rapidly proliferate, providing insights into the underlying molecular mechanisms of CD147 in cancer progression [6, 7]. In addition, the promotion of CD147 in malignant neoplasms strongly depends on its cell surface presentation [8]. Although many studies have reported the role of CD147 in the promotion of tumor migration and invasion [9], proliferation [10], glucose metabolic regulation [11, 12], immune escape [12], and confer resistance to chemotherapeutic drugs [13, 14], there are only few studies that explore the clinicopathological correlation and prognostic relevance of CD147 in BC. The role of CD147 in the regulation of proliferation in BC should be fully elucidated.

For inducing pyroptosis, which is a lytic form of programmed cell death, the pore-forming protein gasdermin D (GSDMD) should be activated by inflammatory caspases through the canonical or noncanonical inflammasome signaling pathway, which in turn mediates the cleavage of GSDMD and the maturation of proinflammatory cytokines, interleukin-1*β* (IL-1*β*), and interleukin-18 (IL-18) [15]. GSDMD pores assist in the leakage of intracellular components into the extracellular environment. A recent study showed that cleavage of gasdermin E (GSDME) by caspase-3 induced pyroptosis in neuroblastoma and melanoma cells, which is followed by treatment with chemotherapeutic agents, such as DNA-binding/modifying compounds (doxorubicin, cisplatin, and actinomycin-D) and topoisomerase inhibitors (topotecan, CPT-11, etoposide, and mitoxantrone) [16]. In addition, another study also showed that the pyroptotic cell death induced by small-molecule inhibitors in lung cancer cells is mediated by caspase-3/GSDME signaling pathway [17]. This implied that the activation of caspase-3/GSDME-dependent pyroptosis might be an alternative therapeutic strategy for cancer treatment. These findings confirmed the vital role of GSDME-mediated pyroptotic cell death in boosting the detrimental effects of chemotherapeutic agents and provided novel insights into the progression of cancer therapy.

The findings of the present study showed significantly upregulated CD147 in BC tissues and cell lines, and its expression was associated with patients' survival. Further studies revealed high expression of CD147 in BC patients with pyroptosis and increased GSDMD expression when compared to healthy controls. These findings revealed that CD147 might contribute to tumor proliferation by regulating GSDMD, which might act as a potential biomarker and therapeutic target for BC treatment.

## 2. Materials and Methods

### 2.1. Cells and Cell Culture

Human urothelial cancer cell lines RT4 and T24 were obtained from the American Type Culture Collection (Manassas, VA, USA). The cells were grown in RPMI-1640 medium (Sigma-Aldrich; Merck KGaA, Darmstadt, Germany) supplemented with 10% (v/v) fetal bovine serum (FBS) (Invitrogen; Thermo Fisher Scientific, Inc., Waltham, MA, USA), penicillin (100 IU/ml), and streptomycin (100 *μ*g/ml).

### 2.2. Antibodies and Reagents

Anti-CD147 (66290-1-lg), Ki-67(27309-1-AP), and anti-GSDMD (20770-1-AP) antibodies were supplied by Proteintech Group (Proteintech, USA). A-tubulin and beta-actin were purchased from Abclonal (Abclonal, China). All reagents were purchased from Sigma (St Louis, MO, USA).

### 2.3. Patients and Biopsies

Based on the Declaration of Helsinki as reflected in a prior approval by the institution's human research committee, this study was conducted in a cohort of patients with BC from 2010 to 2015 and was approved by the Medical Ethical Review Board. A total of 159 patients with BC and 68 healthy controls were included in this study. The detailed information is provided in the Supplementary Materials [Supplementary-material supplementary-material-1]. The bladder tissue was drawn from each patient after obtaining informed consent from them. Written informed consent was given by the caregiver of the patients for using their clinical records, which are not publicly available since the database is currently not anonymous and contains all patient's names; however, they can be provided upon request.

### 2.4. Cell Proliferation Assay

Cell proliferation was measured using a cell counting kit-8 (CCK-8) (Dojindo) as described by Xu et al. [18]. In brief, the cells were seeded into 96-well plate at a concentration of 5 × 10^3^ cells per well. Each well contained 10 *μ*l CCK-8 in 90 *μ*l of culture medium. The cells were incubated for 1 h at 37°C, and the absorbance was measured at 450 nm. The assays were performed in triplicate.

### 2.5. Western Blot Analysis

Western blot analysis was performed as described previously [18]. In brief, the total proteins were collected and subjected to SDS-PAGE and then transferred onto a PDVF membrane. The membranes were blocked for 1 h in tris-buffered saline containing 0.05% Tween-20 and 5% nonfat milk and then were probed with the respective primary antibodies against the target protein overnight at 4°C. The blots were then washed and incubated for 1 h at room temperature with horseradish peroxidase-conjugated anti-rabbit or anti-mouse secondary antibodies. The bands were visualized by using an ECL reagent.

### 2.6. Immunohistochemistry

Immunohistochemistry assay was performed as described by Xu. et al. [18]. In brief, serial cross sections of 4 *μ*m thick were collected and stained immunohistochemically according to the manufacturer's instructions. The samples were deparaffinized, rehydrated, and incubated with fresh 0.3% hydrogen peroxide in methanol for 10 min at 37°C. The sections were then autoclaved for antigen retrieval in citrate buffer at 100°C for 5 min and incubated with indicated antibodies overnight at 4°C. The sections were washed with phosphate-buffered saline (PBS) and incubated with biotinylated anti-rabbit IgG as a secondary antibody for 15 min at 37°C and then with streptavidin-conjugated horseradish peroxidase for 15 min at 37°C (Zymed, Carlsbad, USA). The immune reaction was demonstrated using DAB, and the sections were counterstained with hematoxylin, dehydrated, and then mounted.

### 2.7. Histological Scoring

After the tissues were stained with indicated antibody, five random fields of each section were viewed under a light microscope (Axioskop 40; Zeiss GmbH, Jena, Germany) at ×400 magnification. The sections were independently examined and scored by three investigators who were blinded to the clinical features and outcomes. The expression of GSDMD and CD147 were scored by multiplying the mean signal intensity (on a scale of 0–3: 0, no staining; 1, light staining; 2, moderate staining; and 3, high staining) and the percentage of positively stained tumor cells (on a scale of 0–4: 0, 0%; 1, 0–25%; 2, 26–50%; 3, 51–75%; and 4, 76–100%). The final immunoreactive score was the mean of scores from the three investigators.

### 2.8. Statistical Analysis

All statistical analyses were performed using SPSS software package 22.0 (SPSS Inc, Chicago, IL). The other quantitative data were presented as means ± SD. For tissue array immunohistochemistry analysis, Mann–Whitney *U* test was used to assay the association between CD147 expression and clinicopathological variables in BC tissues and tumor adjacent tissues. A sample *t*-test was used to test the results of mRNA expression, and *p* values less than 0.05 were considered to be significant.

## 3. Results

### 3.1. Patient's Characteristics

A total of 227 subjects (68 healthy controls and 159 patients with BC) were enrolled, and the detailed information of patients are provided in Table 1. Regarding the tumor stage, patients were classified as 80.5% T1 + T2 and 19.5% T3 + T4 stage, respectively. Furthermore, the degree of differentiation in patients were graded as follows: 80 cases (50.3%) are well and moderate, and 79 cases (49.7%) were poorly differentiated, respectively.

### 3.2. CD147 Is Upregulated in Human BC and Correlated with Proliferation in BC Tissues

**Figure 1:**
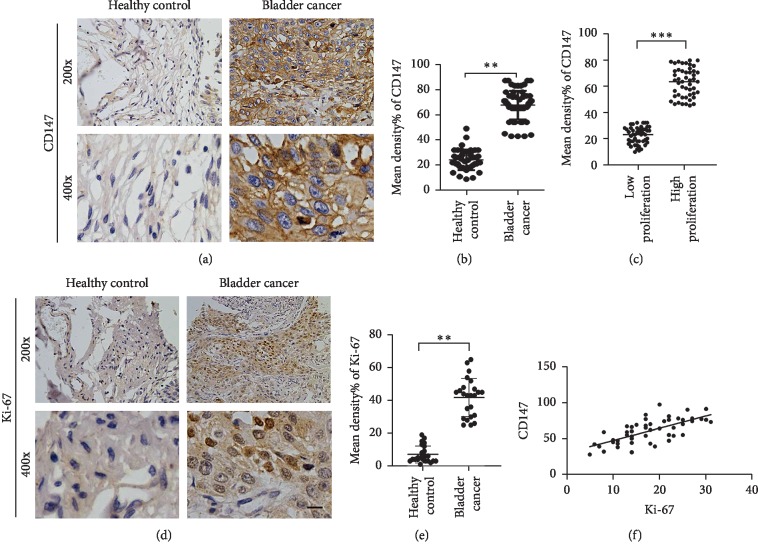
IHC analysis of CD147 and Ki-67 expression in bladder cancer. (a, d) IHC was performed to detect CD147 and Ki-67 expression in clinical sample from bladder cancer and healthy control. Scale bar, 50 m. (b–e) Statistical analysis of mean density of CD147 expression in the indicated group by *t*-test, ^*∗∗*^*p* < 0.01. (f) Clinical association analysis was performed between CD147 and GSDMD.

To explore whether CD147 was involved in the development of BC, the expression of CD147 was compared between BC tissues and normal tissues by immunohistochemistry. The results revealed that CD147 expression in BC tissues (*n* = 159) was increased when compared to normal tissues (*n* = 68) (Figures 1(a) and 1(b)). This suggested that CD147 played a potential role in the development of BC, which was in line with the results from heatmap in TCGA data analysis (Supplementary Figures [Supplementary-material supplementary-material-1]). In addition, based on the tumor staging and dividing into two groups, the results showed that CD147 expression was higher in BC tissues with high proliferation, and stained with Ki-67 (stages III and IV) when compared to those in low-proliferative BCa tissues (stages I and II) (Figures 1(c)–1(e)). Further analysis showed that CD147 expression was positively correlated with Ki-67 (Figure 1(f)). Taken together, these results suggested that the expression of CD147 was closely associated with tumor proliferation in clinical BC tissues.

### 3.3. CD147 Regulated Tumor Proliferation through GSDMD Expression

**Figure 2:**
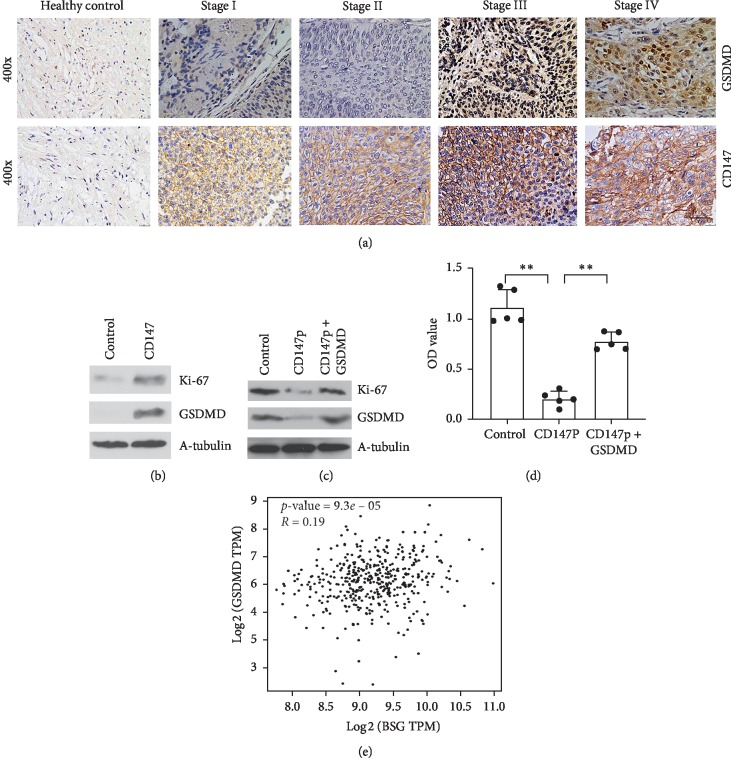
CD147 regulates GSDMD expression in BC. (a) IHC was used to analyze CD147 and GSDMD expression in various stages of bladder cancer tissue, and (b, c) WB was performed to detect the effect of CD147 on GSDMD and Ki-67 expression in the indicated group. (d) CCK-8 assay was employed to detect cell proliferation in the indicated group. (e) TCGA analysis of positive association between CD147 and GSDMD.

To investigate the function of CD147 in tumor proliferation, IHC assay was performed to analyze GSDMD expression, which is considered as a proproliferation of tumor cell [19]. As shown in Figure 2(a), CD147 expression was increased with the tumor stage, and the expression of GSDMD was significantly detected in stages III and IV BC patients. Further results showed that CD147 was positively correlated with GSDMD expression (Figure 2(b)). Treatment with CD147 recombinant protein (BP4745, BOSTER) in RT4 cells, which is a relatively low-grade malignant cell line, significantly contributed to GSDMD expression, leading to increased tumor proliferation as characterized by Ki-67 expression (Figure 2(b)). Blockade of CD147 with peptide (CD147P, bs-0684P, and BIOSS) in T24 cells, which are malignant BC cell lines, decreased the expression of GSDMD and Ki-67, while overexpression of GSDMD reversed the inhibitory effect of CD147 peptide (CD147P) by Ki-67 expression (Figure 2(c)). In addition, the results from CCK-8 assay also indicated that overexpression of GSDMD significantly overcame the inhibitory effect of CD147 peptide on cell proliferation (Figure 2(d) and [Supplementary-material supplementary-material-1]). Furthermore, CCK-8 assay revealed that TCGA analysis from BC showed that CD147 was associated with GSDMD in patients with BC (Figure 2(e)). These findings suggested that CD147 had an influence on cell proliferation in GSDMD-dependent pathway.

### 3.4. CD147 Is an Unfavorable Prognostic Factor

**Figure 3:**
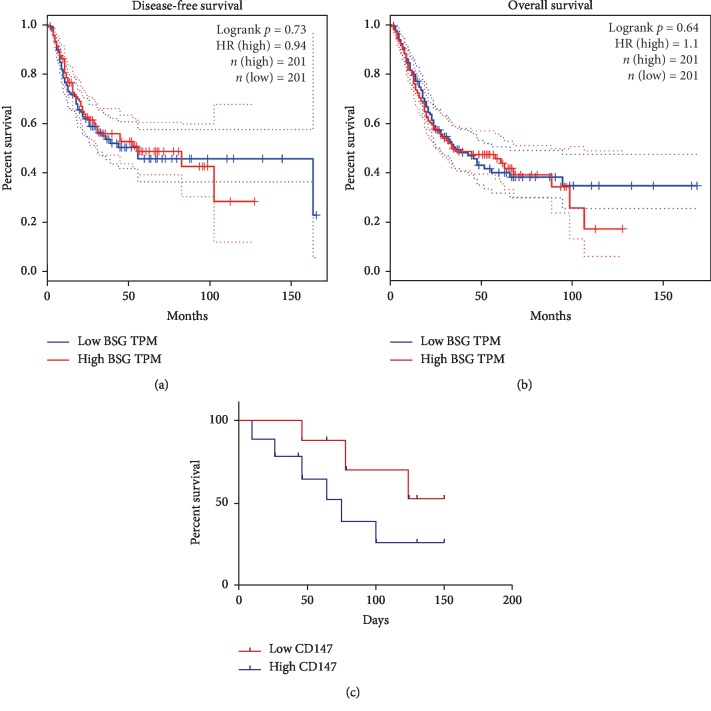
CD147 is a worse prognostic factor for mortality in BC patients. a and b represent the Kaplan–Meier analysis of DFS (a) and OS (b) in BC patients according to CD147 expression from TCGA dataset. (c) Survival rate was analyzed in 159 subjects classified into two groups based on CD147 expression, *p*=0.041.

The results have shown that CD147 was increased and closely associated with differentiation in BC tissues. However, TCGA data analysis also showed poorer disease-free survival (DFS) in patients with higher expression of CD147 (Figure 3(a)), while a slight shorter overall survival (OS) was observed in patients with high CD147 expression when compared to patients with low CD147 expression. To further discover the relationship between CD147 expression and clinical prognosis, survival rate by analyzing of 159 subjects was performed. The findings showed that patients with enriched CD147 had a shorter survival rate when compared to those with weakened CD147 expression (Figure 3(c)). These conflicting results might attribute to the limitation of number of subjects, but requires confirmation. Taken together, these findings implied that CD147 was an unfavorable prognostic factor for mortality in patients with BC.

## 4. Discussion

Few studies showed a pivotal role of CD147 in tumor recurrence and metastasis, leading to difficulties in the completion of tumor treatments in time [20]. In this study, CD147 promoted cell proliferation in BC through upregulating GSDMD expression. Treatment with CD147 in BC significantly increased GSDMD, leading to cell proliferation by CCK-8 assay, while blocking CD147 with peptide reduced cell proliferation. Interestingly, overexpression of GSDMD drastically reversed the inhibitory effect of CD147 peptide on cell proliferation. These findings implied a novel role of CD147 in cell proliferation.

In line with this, a meta-analysis study conducted by Li et al. also showed that CD147 expression is closely associated with prognostic and clinicopathological characteristics of BC [21]. CD147 forms complexes with *α*3*β*1 and *α*6*β*1 integrins by cell-cell contact, which in turn promotes tumor invasion by inducing matrix metalloproteinase (MMP) synthesis via a focal adhesion kinase- (FAK-) PI3K signaling pathway [22]. In addition, CD147 is an inducer of extracellular MMP and suggested to play an important role in cell differentiation due to developmental defects observed in the knockout mice [23–26]. These include active differentiation of basal cells in the epithelium, which show a strong and constitutive expression of CD147, whereas a weak expression in the superficial terminally differentiated cells [27]. Hence, these studies indicated the importance of CD147 function in tumor development.

Although a number of studies have showed that overexpression of CD147 is associated with patients' survival in BC [28, 29], some studies have limitations, such as the involvement of small-sized cohort or lack of examining mechanism. In this study, the relationship of CD147 with patients' prognosis and survival was detected in a large cohort of BC that are grouped into different stages. The results revealed that CD147 expression was significantly upregulated in BC, which in turn were correlated with tumor proliferation. Our findings also supported that high CD147 expression might be a biological parameter for poor prognosis and survival.

Most of the previous reports studied the mechanisms of CD147 induction of tumor progression by promoting MMP expression or monocarboxylate transporters (MCT) interaction [30, 31], and therefore other markers have rarely been explored. GSDMD belongs to gasdermin (GSDM) family, and acts as an inducer of pyroptosis [32]. Some studies have demonstrated that GSDMD is required for the activation of effector T-cell responses in cancer cells [33]. However, CD147 plays a negative role in regulating T-cell-mediated immune responses [34]. Direct evidence for the crosslink between CD147 and GSDMD in cancer is lacking. In this study, a relationship of CD147 and GSDMD in BC was also examined. Blocking CD147 with peptide leads to decreased GSDMD expression, resulting in changes in cell proliferation marker expression, which is labeled with Ki-67. In addition, overexpression of GSDMD significantly reversed the inhibitory effect of CD147 peptide on cell proliferation. However, further work requires clarification on how CD147 regulates GSDMD expression.

## 5. Conclusion

In conclusion, CD147 is increased in patients with BC and showed association with GSDMD expression, leading to increased tumor proliferation. Furthermore, overexpression of CD147 suggested poor prognosis and survival, which in turn might help to determine its significance as a new target in the treatment of BC.

## Figures and Tables

**Table 1:** The characteristic of patients with bladder cancer.

Variables	Bladder cancer
Age (years)	
>65	53 (28%)
≤65	106 (72%)

Sex	
Male	119 (63%)
Female	40 (37%)

T stage	
T1 + T2	128 (80.5%)
T3 + T4	31 (19.5%)

Differentiation	
Well/moderately	80 (50.3%)
Poorly	79 (49.7%)

CD147 expression	
Low expression	90 (56.6%)
High expression	69 (43.4%)

## Data Availability

The data used to support the findings of this study are available from the corresponding author upon request.

## Supplementary Materials

Supplementary Materials Figure 1S: TGCA analysis of CD147: (A-B) CD147 expression is increased in BC; (C) Cell proliferation assay via CCK-8 kit.
