# Supplementary figures and images for: CD147 Expression Is Associated with Tumor Proliferation in Bladder Cancer via GSDMD
Source: Biomed Res Int. 2020 Feb 20;2020:7638975. doi: 10.1155/2020/7638975 (PMC7054768; doi:10.1155/2020/7638975)

**Fig.1s**

**A**

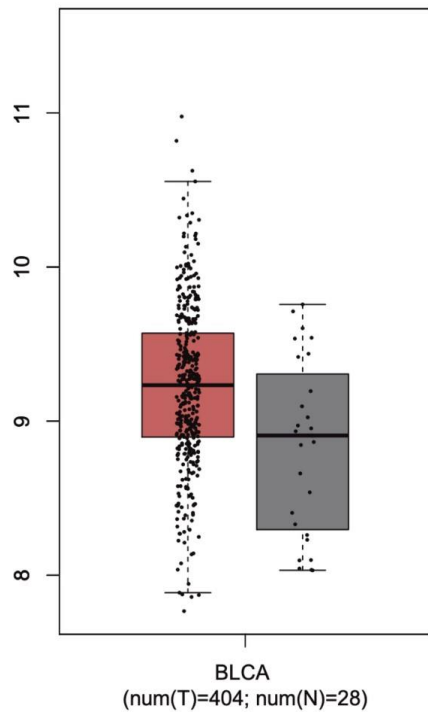

**B**

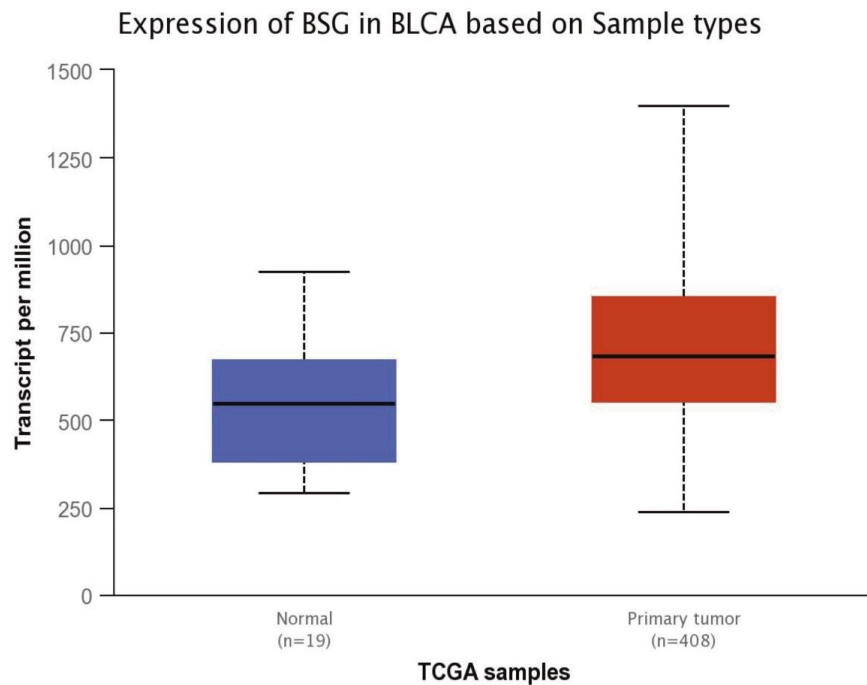

Supplement: Supplementary Materials — Figure 1S: TGCA analysis of CD147: (A-B) CD147 expression is increased in BC; (C) Cell proliferation assay via CCK-8 kit. [file 7638975.f1.pdf]
